# Draft Genome Sequence of a Pseudomonas aeruginosa Sequence Type 3351 Strain Exhibiting High-Level Resistance to Polymyxins in a Pediatric Patient with Cystic Fibrosis in Mexico

**DOI:** 10.1128/MRA.01261-19

**Published:** 2020-01-09

**Authors:** Roberto Rosales-Reyes, Fernanda Esposito, Bruna Fuga, Louise Cerdeira, Catalina Gayosso-Vázquez, José L. Lezana-Fernández, Ricardo Lascurain, Miguel A. Valvano, Nilton Lincopan, José I. Santos-Preciado

**Affiliations:** a Unidad de Medicina Experimental, Facultad de Medicina, Universidad Nacional Autónoma de México, Mexico City, Mexico; b Iberoamerican Network for Combating Antimicrobial Resistance (INCAR)‡; c Department of Clinical Analysis, School of Pharmaceutical Sciences, Universidade de São Paulo, São Paulo, Brazil; d Department of Microbiology, Institute of Biomedical Sciences, Universidade de São Paulo, São Paulo, Brazil; e Laboratorio de Fisiología Pulmonar, Hospital Infantil de México Federico Gómez, Mexico City, Mexico; f Departamento de Bioquímica, Facultad de Medicina, Universidad Nacional Autónoma de México, Mexico City, Mexico; g The Wellcome-Wolfson Institute for Experimental Medicine, Queen’s University Belfast, Belfast, United Kingdom; University of Arizona

## Abstract

Here, we present the draft genome sequence of a Pseudomonas aeruginosa isolate (strain CF16053) belonging to a novel sequence type (ST), ST3351, isolated from a pediatric patient with cystic fibrosis (CF). CF16053 shows high-level resistance to polymyxins associated with mutations in the *pmrB* gene. Biofilm, pyoverdine, exotoxin A, and type III secretion system (T3SS) genes were identified.

## ANNOUNCEMENT

Respiratory infections are the main cause of morbidity and mortality in patients with cystic fibrosis (CF), in whom Pseudomonas aeruginosa remains a leading pathogen (1, 2). Genomic investigation of antibiotic-resistant P. aeruginosa strains is essential for better understanding the molecular epidemiology and evolution of this pathogen, as well as for improving clinical outcomes in CF patients (3, 4). In this study, we report the draft genome sequence of a P. aeruginosa strain exhibiting high-level resistance to polymyxins in a pediatric patient with CF in Mexico.

P. aeruginosa strain CF16053 was isolated from a sputum culture from a 5-year-old male with CF. The sputum was inoculated onto MacConkey, chocolate, blood (Dibico), and cetrimide (Becton, Dickinson) agar plates. Inoculated plates were incubated aerobically at 37°C for up 48 h. Initial bacterial identification was performed using standard microbiological methods, and the species were confirmed with the API 20NE (bioMérieux SA) system. All samples were stored at −70°C until they were analyzed. Strain CF16053 was subcultured once before genomic analysis was performed. Identification and antibiotic susceptibility were determined by the Vitek 2 platform, whereas colistin and polymyxin B MICs were determined by microdilution following 2019 CLSI guidelines (5). The strain showed resistance to piperacillin-tazobactam (MIC > 128 μg/ml), colistin (MIC > 128 μg/ml), and polymyxin B (MIC > 128 μg/ml).

Total DNA from an isolated colony was extracted using the PureLink quick gel extraction kit (Life Technologies, CA). DNA quality and quantity were evaluated by agarose gel electrophoresis and by using a Qubit 2.0 fluorometer (Life Technologies). A genomic library was constructed using a Nextera DNA Flex library preparation kit, with subsequent sequencing by the MiSeq platform (300-bp paired-end reads; Illumina, Inc., San Diego, CA). The resistome and virulome were obtained using ResFinder version 3.2 (6), the Comprehensive Antibiotic Resistance Database (CARD) (7), and VFanalyzer (8), respectively, whereas the multilocus sequence type (MLST) was predicted using MLST version 2.0 (9).

A total of 1,952,320 paired-end reads were generated with 94.46× coverage and *de novo* assembled into 52 contigs using SPAdes version 3.9.0 (10). Default parameters were used for all software unless otherwise specified. Quality filters were applied, and a Phred quality score of 20 was used. The sequence assembly was curated using Geneious version R9 (Biomatters Ltd., New Zealand) and submitted for annotation using NCBI Prokaryotic Genome Annotation Pipeline (PGAP) v.3.2 (11). The *N*_50_ value obtained was 425,394 bp. The genome size was calculated as 6,174,571 bp with a GC content of 66.4%, and the genome comprised 5,780 protein-coding sequences. In addition, 5,905 complete genes, 57 tRNAs, 3 rRNAs, 4 noncoding RNAs (ncRNAs), and 61 pseudogenes were identified.

The P. aeruginosa MLST database indicated a novel sequence type, where the different alleles of each gene were numbered *acsA17*, *aroE5*, *guaA11*, *mutL3*, *nuoD3*, *ppsA4*, and *trpE10*; it was therefore assigned to sequence type 3351 (ST3351) (12). Resistome analysis revealed that CF16053 harbored resistance genes to β-lactams (*bla*_OXA-488_ and *bla*_PAO_), phenicols (*catB7*), fosfomycin (*fosA*), and aminoglycosides [*aph(3′)-llb*]. Additionally, mutations in *pmrB* (T90A, H140Y, G211R, G213S, T215A, N250D, and V344A) and *pmrA* (L71A) genes that contribute to high-level polymyxins resistance (13, 14) were also identified.

Virulome analysis identified genes related to biofilm formation (quorum sensing genes *lasA* and *ptxR*) and genes associated with alginate synthesis (*algG*, *algI*, *alg8*, *algE*, *algA*, *algX*, *algK*, *algF*, *algD*, *algJ*, *alg44*, *algL*, and *algB*). Furthermore, siderophore pyoverdine synthesis (*pvdA*, *pvdD*, *pvdE*, *pvdF*, *pvdG*, *pvdL*, *pvdN*, *pvdO*, *pvdQ*, *pvdP*, and *pvdS*), exotoxin A (*toxA*), and type III secretion system (T3SS) (*exoS* and *exoT*) genes were also identified.

In summary, we report for the first time the draft genome sequence of a novel ST3351 (determined by MLST) P. aeruginosa strain displaying high-level resistance to polymyxins that was obtained from a child with CF in Mexico. These data could contribute to a better understanding of acquired resistance in P. aeruginosa lineages infecting people with CF.

### Data availability.

The genome sequence of Pseudomonas aeruginosa strain CF16053 has been deposited in GenBank under accession number VTFM00000000 (SRA accession number PRJNA562177).
